# Associations between abnormal spontaneous neural activity and clinical variables, eye movements, and event-related potential indicators in major depressive disorder

**DOI:** 10.3389/fnins.2022.1056868

**Published:** 2023-01-11

**Authors:** Qinqin Zhang, Xiaoling Li, Haohao Yan, Yun Wang, Yangpan Ou, Yang Yu, Jiaquan Liang, Hairong Liao, Wanting Wu, Xiancong Mai, Guojun Xie, Wenbin Guo

**Affiliations:** ^1^Department of Psychiatry, The Third People's Hospital of Foshan, Foshan, Guangdong, China; ^2^Department of Psychiatry, National Clinical Research Center for Mental Disorders, The Second Xiangya Hospital of Central South University, Changsha, Hunan, China

**Keywords:** major depressive disorder, resting-state functional magnetic resonance imaging, fractional amplitude of low-frequency fluctuations, sensorimotor network, support vector machine

## Abstract

**Background:**

This study aimed to investigate the correlations between abnormal spontaneous neural activity measured with fractional amplitude of low-frequency fluctuations (fALFF) and clinical variables, eye movements, and event-related potential indicators in patients with major depressive disorder (MDD).

**Methods:**

We recruited 42 patients with MDD and 42 healthy controls (HCs) and collected their clinical variables, eye movement, event-related potential, and resting-state functional magnetic resonance imaging (rs-fMRI) data. The fALFF, support vector machine (SVM), and correlation analysis were used to analyze the data.

**Results:**

The results of the study showed that the fALFF values of the sensorimotor network, including the right middle temporal gyrus, right cerebellar Crus2, left occipital gyrus, and left middle temporal gyrus, were significantly higher compared to HCs. Correlation analysis showed that the abnormal fALFF value of the right cerebellar Crus2 was inversely correlated with the active coping scores of the Simplified Coping Style Questionnaire in the patients (r = −0.307, *p* = 0.048). No correlation was observed between abnormal fALFF values and other clinical symptoms, neuropsychological tests, eye movements, and event-related potential-related indicators in patients with MDD. fALFF values in the left middle temporal gyrus could be used to distinguish patients with MDD from HCs with an accuracy of 78.57%.

**Conclusions:**

Patients with MDD exhibited enhanced spontaneous neural activity in the sensorimotor network. No associations were found between abnormal spontaneous neural activity and clinical variables, eye movements, and event-related potential related indicators in MDD.

## Introduction

Major depressive disorder (MDD) is a common mental disease in the clinic, with depressive mood, lack of interest or pleasure, and decreased energy as the main clinical manifestations. Severe patients may experience suicidal ideations and behaviors. The incidence of MDD is high, with the latest data from the World Health Organization reporting that the global incidence of MDD is 3.1% (Smith, 2014). As a chronic disease, MDD is characterized by recurrent episodes. Studies have shown that near three-quarters of patients who have experienced a major depressive episode will experience another depressive episode, and the risk of relapse gradually increases with the increase in the number of episodes (McLaughlin, 2011). In addition, the suicide risk of patients with MDD is much higher than that of normal people (Burcusa and Iacono, 2008; Miret et al., 2013). MDD not only leads to the impairment of social function, but also increases the risk of diabetes, stroke, and hypertension, which leads to an increase in the suicide rate and disability rate, and brings serious consequences to the family and society (McLaughlin, 2011; Miret et al., 2013; Roca et al., 2019).

However, the underlying neurobiological mechanism of MDD remains to be explored. Resting-state functional magnetic resonance (rs-fMRI) technology has been widely used in the study of MDD. The amplitude of low-frequency fluctuations (ALFF) is a commonly used rs-fMRI analysis method to assess regional spontaneous activity, which was proposed by Zang et al. (2007). However, ALFF is sensitive to physiological noise. Thus, Zou et al. proposed a fractional ALFF (fALFF) method, a modified ALFF method, which can measure the local fluctuation of neuronal activity, and has higher sensitivity than the ALFF. Therefore, the deviation of non-specific physiological signal components is smaller (Zou et al., 2008). A large amount of neuroimaging evidence has demonstrated abnormal brain function and structure in patients with MDD in the resting state (Lai and Wu, 2015; Gray et al., 2020; Wu et al., 2020; Chase et al., 2021; Ebneabbasi et al., 2021). A study by Wang et al. (2012) involving 18 first-episode, untreated patients with MDD and 18 healthy controls (HCs) found that patients with MDD had significantly higher fALFF values in the right precentral gyrus, right inferior temporal gyrus, bilateral fusiform gyrus, and bilateral anterior and posterior cerebellar lobes. However, it was significantly decreased in the left dorsolateral prefrontal cortex, bilateral medial orbitofrontal cortex, bilateral middle temporal gyrus, left inferior temporal gyrus, and right inferior parietal lobule. A similar study by Shen et al. (2014), however, found decreased fALFF in the right angular gyrus, left middle temporal gyrus, left superior temporal gyrus, right putamen, right precuneus, and right superior temporal gyrus, without an increase in fALFF values in the patients. Liu et al. (2013) unexpectedly found a significant decrease in fALFF in the right posterior lobe of the cerebellum in patients with MDD with depression, whereas Guo W. et al. (2013) found a significant increase in fALFF values in the left Crus I and the left cerebellar lobule VI, suggesting that there may be a cerebellar compensatory response in patients with MDD. In general, these studies have produced different results due to relatively small sample sizes, study designs, and analysis methods. Previous studies have shown that people with MDD have abnormalities in multiple brain regions including the frontal, temporal, parietal, and cerebellar regions (Wang et al., 2012; Guo W. B. et al., 2013; Liu et al., 2014).

Abnormal regional activity may be related to the cognitive impairment of MDD. Most patients with MDD have impaired cognitive function, which is an important reason for the persistence of depressive symptoms (Dehn and Beblo, 2019). Previous studies have found obvious cognitive dysfunction in patients with MDD, such as concentration (Srisurapanont et al., 2018; Zainal et al., 2019), memory decline (Li et al., 2018), executive force down (Paelecke-Habermann et al., 2005), and spiritual movement disorders (Mondal et al., 2007). At present, the cognitive dysfunction of MDD is mainly explored from the aspects of neuroelectrophysiology, neuroimaging, and neuropsychological tests. Neuroelectrophysiological studies on cognitive dysfunction in MDD mostly focus on P300. Some studies have proved that prolonged latency and lower amplitude of P300 can predict the severity of MDD and reflect cognitive impairment of the patients (Tripathi et al., 2015; Nan et al., 2018). Studies have shown that P300 amplitude is related to information processing function, while latency is related to classification processing (Kaustio et al., 2002). However, previous imaging studies have shown that abnormal working memory in patients with MDD is related to frontal, temporal, parietal, and subcortical activation (Wang et al., 2015; Yuksel et al., 2018). The impaired executive function may be related to abnormal functional connectivity (FC) in the prefrontal cortex (Liu et al., 2020). In addition, other studies suggest that the cortical-thalamo-striatal circuit (OSCT) may be involved in the impairment of cognitive function in patients with MDD (Li et al., 2017; Wang et al., 2018).

Although there are many studies on MDD in the past, the indicators used in these studies are relatively single. In this study, we aimed to explore the neuropathological mechanism of MDD by combining neuropsychological tests, eye movements, event-related potentials, and rs-fMRI. We hypothesized that patients with MDD would show changes in fALFF values in certain brain regions, especially frontal, temporal, parietal, and cerebellar regions, which may be a potential indicator to distinguish patients with MDD from HCs. In addition, we hypothesized that abnormal fALFF values were related to clinical variables, eye movements, and event-related potentials in the patients.

## Materials and methods

### Participants

From September 2020 to April 2022, we recruited 46 patients with initial or recurrent MDD (32 first-episode patients, 10 recurrent patients) and 44 HCs matched for sex, and years of education from The Third People's Hospital of Foshan. The patient version of the Structured Clinical Interview for Diagnostic and Statistical Manual of Mental Disorders-5 (DSM-5) was used to diagnose MDD. The recurrent patients had stopped taking antidepressants for at least 2 weeks. Depressive and anxiety symptoms, personality characteristics, social function, social support, coping style, and psychological cognitive function of the subjects were measured by Hamilton Depression Scale (HAMD-24), Hamilton Anxiety Scale (HAMA), Eysenck Personality Questionnaire (EPQ), Social Disability Screening Schedule (SDSS), Social Support Revalued Scale (SSS), Simplified Coping Style Questionnaire (SCSQ), Repeatable Battery for the Assessment of Neuropsychological Status (RBANS), and Wisconsin Card Sorting Test (WCST). All individuals were aged 18–60 years and right-handed. Two groups had the same exclusion criteria as follows: (1) history of serious physical illness or substance abuse such as alcohol; (2) severe physical disability, unable to complete the follow-up study; (3) Comorbid other severe psychiatric disorders, intellectual disability, dementia, and severe cognitive impairment; (4) Undergoing or being prepared for additional clinical studies.

The study was confirmed by the Research Ethics Committee of the Third People's Hospital of Foshan. All participants signed written informed consent.

### Exploratory eye movement data acquisition

The data were obtained with a Dekang DEM-2000 eye movement detector made in Shanghai. The subjects were asked to sit comfortably in a chair and look at a small screen in front of them. The distance between their eyes and the screen was 25 cm, and the Angle of their eyes moving from the left side of the screen to the right side was 33°. The first S-shaped pattern (S) was first displayed on the screen for 15 s and the subjects were asked to observe carefully. The instrument automatically recorded the gaze points within 15 s and counted them as the number of eye fixation (NEF). Then, the second and third S-shaped patterns (S_2_, S_3_) were displayed on the screen, which were slightly different from the first figure. Each pattern lasted for 15 s. Subjects were asked to observe carefully and repeatedly asked “what is the difference between the two patterns and the first figure” until the subjects answered “there is no difference”. Then the gaze points in seven regions (only one point was counted in each region) were recorded for a total of 5 s, which was used as the responsive searching score (RSS). The instrument can automatically record the trajectory of eye movements, the data are automatically analyzed by the computer, and the whole process can be played back for future reference. In EEM analysis, NEF refers to the total number of gaze points in 15 s when the eye fixates on the S-pattern, and a gaze point refers to the eye's gaze time exceeding 200 ms to a certain point (the movement of the eyeball is within 2°). The RSS score is divided into seven areas of S_2_ or S_3_, and the number of areas of eye fixation is measured by the instrument for a total of 5 s. The subject's eye fixation on a certain area is scored one point, regardless of how many times. Therefore, the maximum RSS score for each image is seven, and the maximum RSS total score for S_2_ and S_3_ is 14. Abnormal criteria: NEF < 30 and/or RSS < 4 were considered abnormal.

### Event-related potential data acquisition

ERP data were acquired using a Nihon Kohden MEB-9402C electromyography evoked potential meter. The subjects took a seated position, remained relaxed, and tried to concentrate. The electrode position followed the 10/20 standard of the International Electroencephalography Association. The central Cz point was used as the recording electrode, the right ear M2 point was used as the reference electrode, and the ground was placed in the middle of the forehead FPz, and set electrode impedance < 5 K*Ω*, filter 0.5–100 Hz, and analysis time 1,000 ms. Using the classic “Oddball” auditory stimulation mode, the stimulation is performed at a frequency of 1 time/s, the duration is 10 ms, and the sensitivity is 5 μV. Both the low-frequency filter and the high-frequency filter were band-pass superimposed 200 times, and the detection was performed by triggering and stimulating the two systems. The parameters of non-target stimulation were set to 80% probability, 70 dB intensity, and 1,000 Hz frequency; the parameters of target stimulation were 20% probability, 90 dB intensity, and 2,000 Hz frequency. The two frequencies were randomly interspersed, and each case was repeated twice, taking the average value. The participant was told to count for the target stimulus, and the non-target stimulus was not used as a response. If the subject's hit rate is < 80%, the test is invalid. The latency of N100, P200, N200, and P300 waves were recorded, respectively.

### Imaging data acquisition

A GE 3.0 T scanner (GE 3.0 T Signa Pioneer) was used to acquire images. Participants were asked to remain stationary, close their eyes, and remain awake. To minimize the influence of scanner noise and head motion, soft earplugs and foam pads were used. Scanning parameters were: repetition time/echo time = 2,000/30 ms, 36 slices, 64 × 64 matrix, 90° flip angle, 22 cm field of view, 4 mm slice thickness, no gap, and 250 volumes (500 s).

### Data preprocessing

DPARSF software package was used to preprocess the acquired imaging data in MATLAB (http://www.mathworks.com) (Chao-Gan and Yu-Feng, 2010). To acclimate the participants to the scanning environment and ensure a stable signal, the first 10 images were discarded. Slice timing and head movement correction were performed on the remaining 240 volumes. Each participant's head movement should be <2° in any angular rotation and <2 mm displacement in the *x*-, *y*-, or *z*-axis. The corrected images were then normalized to the standard Montreal Neurological Institute (MNI) space and resampled to 3 × 3 × 3 mm^3^ to perform linearly detrended and band-pass filtering (0.01–0.08 Hz).

### fALFF calculation

DPARSF software package was used to calculate fALFF. Zou et al. (2008) described the process of obtaining fALFF maps. First, each voxel's time series was converted to the frequency domain using a fast Fourier Transform and acquired the power spectrum. The processed scans were then spatially smoothed with an 8 mm full width at half maximum Gaussian kernel. After that, the square root was calculated at each frequency of the power spectrum to obtain the mean square root within the 0.01–0.08 Hz frequency band for each voxel. Finally, each voxel was normalized by dividing the fALFF of each voxel by the global average fALFF value within the brain mask.

### Statistical analysis

SPSS version 25.0 was used to analyze the data in this study. The difference in sex between the two groups was analyzed using a Chi-square test. For continuous variables such as age, years of education, and clinical scales, the two-sample *t*-tests were used. The significance level was set as *p* < 0.05.

Image data were analyzed by the DPARSF software package. Two-sample *t*-tests were performed for each normalized fALFF map. The significance level was set at *p* < 0.05 and multiple comparisons corrected with the Gaussian Random Field (GRF) theory (voxel significance: *p* < 0.001, cluster significance: *p* < 0.05). Age, sex, education level and mean framewise displacement (FD) were used as covariates to minimize the potential effects of these variables.

Mean fALFF values were extracted from abnormal brain regions showing significant differences between patients with MDD with MDD and HCs for further correlation analysis. Pearson or Spearman correlation analyses were used to analyze the association between fALFF values and clinical variables in patients and HCs. The significance level was set at *p* < 0.05 (corrected according to the Bonferroni correction).

### SVM analysis

LIBSVM software package (Chang and Lin, 2011) was used for classification analysis to explore whether abnormal fALFF values could be used as diagnostic imaging biomarkers to distinguish patients from HCs. To obtain the highest sensitivity and specificity, we adopted a “leave-one-out” cross-validation approach (Liu et al., 2018).

## Results

### Demographic and clinical data

A total of 46 patients with MDD with MDD and 44 HCs were recruited for this study. Four patients with MDD and two HCs were excluded due to excessive head movement. Therefore, the final analysis included 42 patients with MDD and 42 HCs. The demographic and clinical data of participants were shown in Table 1. There were significant differences in age (*p* = 0.01) between the patient group and the HC group, whereas there was no significant difference in gender and years of education. There were significant differences in HAMD (*p* < 0.001), HAMA (*p* < 0.001), Extraversion (E) (*p* = 0.003), Neuroticism (N) (*p* < 0.001), Lie (L) (*p* < 0.001), SDSS (*p* < 0.001), SSS (*p* < 0.001), SCSQ subscale scores (*p* < 0.001, *p* = 0.001), NEF (*p* < 0.001), RSS (*p* = 0.025), N200 (*p* = 0.038), and P300 (*p* = 0.012) between the two groups, but no significant difference in SCSQ total scores. There was no significant difference between the two groups in WCST, RBANS, N100, and P200.

**Table 1:** Characteristics of participants.

**Variables**	**Patients (*n* = 42)**	**Controls (*n* = 42)**	***p*** **-value**
Age (years)	26.43 ± 10.79	35.14 ± 12.54	0.001^a^
Sex (male/female)	15/27	18/24	0.503^b^
Years of education (years)	13.48 ± 2.48	12.62 ± 3.72	0.218^a^
HAMD	24.80 ± 7.22	2.55 ± 3.54	< 0.001^a^
HAMA	16.60 ± 5.70	2.03 ± 2.69	< 0.001^a^
**EPQ**			
P	51.20 ± 8.19	47.23 ± 12.68	0.092^a^
E	40.16 ± 11.42	48.72 ± 13.93	0.003^a^
N	68.37 ± 9.19	45.08 ± 10.01	< 0.001^a^
L	44.92 ± 11.30	56.93 ± 11.62	< 0.001^a^
SDSS tosal score	7.07 ± 2.42	0.02 ± 0.15	< 0.001^a^
**SSS**			
Total score	28.60 ± 8.71	43.14 ± 9.33	< 0.001^a^
Objective support score	7.45 ± 3.41	10.93 ± 2.85	< 0.001^a^
Subjective support score	14.50 ± 5.23	23.36 ± 5.91	< 0.001^a^
Utilization of support	6.64 ± 2.12	8.86 ± 2.05	< 0.001^a^
**SCSQ**			
Total score	26.83 ± 8.42	29.90 ± 9.43	0.119^a^
Active coping	16.50 ± 6.19	22.90 ± 7.35	< 0.001^a^
Negative coping	10.33 ± 4.24	7.00 ± 4.35	0.001^a^
**WCST**			
CC	5.00 ± 1.21	5.26 ± 1.23	0.328^a^
RA	46.05 ± 2.71	44.00 ± 4.02	0.008^a^
RC	34.55 ± 5.58	34.93 ± 3.58	0.711^a^
RE	11.50 ± 6.88	9.02 ± 6.21	0.087^a^
RP	3.57 ± 4.94	2.14 ± 3.33	0.124^a^
RPE	1.79 ± 2.85	0.81 ± 1.45	0.052^a^
**RBANS**			
Immediate memory	42.15 ± 10.40	42.83 ± 11.69	0.781^a^
Visuospatia/ constructional	18.73 ± 2.16	17.90 ± 2.29	0.100^a^
Language	17.60 ± 4.43	18.57 ± 4.20	0.311^a^
Attention	60.60 ± 14.11	64.81 ± 16.09	0.212^a^
Delayed memory	48.33 ± 9.65	49.60 ± 10.66	0.574^a^
**EEM**			
NEF	21.11 ± 5.95	27.52 ± 4.32	< 0.001^a^
RSS	3.71 ± 1.51	4.60 ± 1.62	0.025^a^
**ERP**			
N100	103.73 ± 15.35	107.86 ± 34.86	0.508^a^
P200	174.53 ± 21.59	177.62 ± 23.66	0.545^a^
N200	230.21 ± 31.52	211.79 ± 44.69	0.038^a^
P300 (ms)	309.74 ± 24.20	287.10 ± 50.80	0.012^a^

HAMD, Hamilton Depression Rating Scale; HAMA, Hamilton Anxiety Rating Scale; EPQ, Eysenck Personality Questionnaire; P, psychoticism; N, neuroticism; E, extraversion; L, lie; SDSS, Social Disability Screening Schedule; SSS, Social Support Revalued Scale; SCSQ, Simplified Coping Style Questionnaire; WCST, Wisconsin Card Sorting Test; CC, categories completed; RA, responses answer; RC, correct responses; RE, errors responses; RP, perseverative responses; RPE, perseverative responses errors; RBANS, Repeatable Battery for the Assessment of Neuropsychological Status; EEM, exploratory eye movement; NEF, number of eye fixation; RSS, responsive search score; ERP, event related potential.

a The *p*-values were obtained by two samples *t*-tests.

b The *p*-value for sex distribution was obtained by a Chi-square test.

### fALFF analysis in patients with MDD and HCs

**Figure 1:**
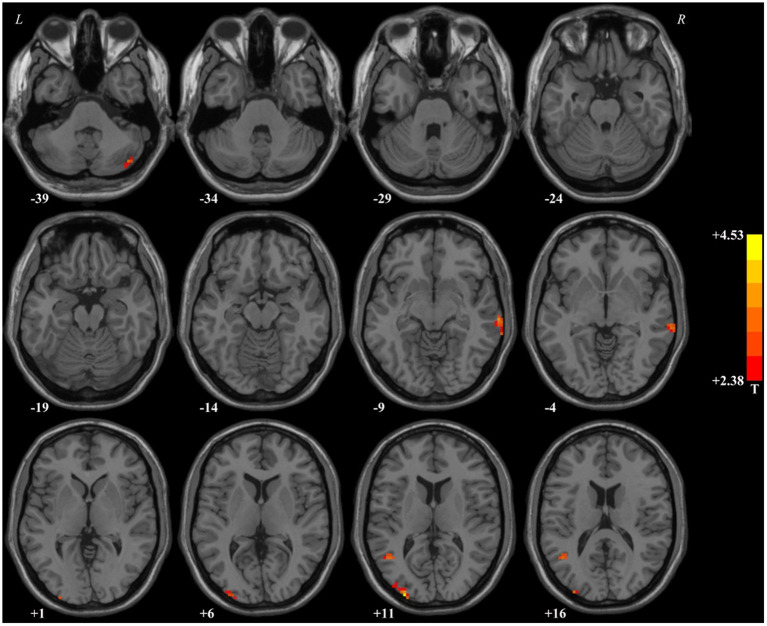
Brain regions with significant difference in the fALFF values between patients with MDD and healthy controls. In comparison with healthy controls, patients with MDD had higher fALFF values in the right middle temporal gyrus, right cerebellar Crus2, left occipital gyrus, and left middle temporal gyrus. fALFF, fractional amplitude of low-frequency fluctuations.

In comparison with HCs, patients with MDD had higher fALFF values in the right middle temporal gyrus, right cerebellar Crus2, left occipital gyrus, and left middle temporal gyrus. Detailed information is provided in Table 2 and Figure 1.

**Table 2:** Regions with abnormal fALFF values in patients with MDD.

**Cluster location**	**Peak (MNI)**			**Number of voxels**	***T*** **-value**
	***x***	***y***	***z***		
Right middle temporal gyrus	69	−33	−6	33	3.6268
Right cerebellar crus2	42	−75	−39	30	3.3639
Left occipital gyrus	−30	−96	12	26	4.5263
Left middle temporal gyrus	−48	−57	12	26	3.4134

MDD, major depressive disorder; MNI, Montreal Neurological Institute; fALFF, fractional amplitude of low-frequency fluctuations.

### SVM analysis

**Figure 2:**
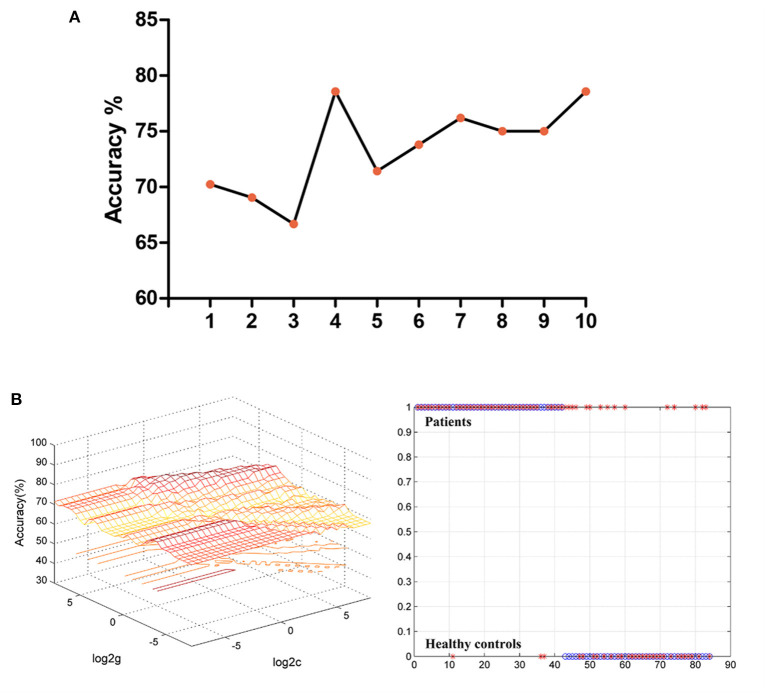
**(A)** The accuracy of classification of different imaging features used as support vector in SVM analyses. The “1” represented the fALFF values of the right middle temporal gyrus; The “2” represented the fALFF values of the right cerebellar Crus2; The “3” represented the fALFF values of the left occipital gyrus; The “4” represented the fALFF values of the left middle temporal gyrus; The “5” represented the fALFF values of the right middle temporal gyrus and right cerebellar Crus2; The “6” represented the fALFF values of the right middle temporal gyrus and left occipital gyrus; The “7” represented the fALFF values of the right middle temporal gyrus and left middle temporal gyrus; The “8” represented the fALFF values of the right cerebellar Crus2 and left occipital gyrus; The “9” represented the fALFF values of the right cerebellar Crus2 and left middle temporal gyrus. The “10” represented the fALFF values of the left occipital gyrus and left middle temporal gyrus. **(B)** The results of classification based on the fALFF values of the left middle temporal gyrus. The accuracy = 78.57%, sensitivity = 92.86%, and specificity = 64.29%. SVM, support vector machine; fALFF, fractional amplitude of low-frequency fluctuations.

Figure 2A shows the accuracy of distinguishing patients with MDD from HCs based on the fALFF values of the four detected brain regions and the combination of these clusters. Based on the fALFF values in the left middle temporal gyrus, the accuracy was 78.57%, the sensitivity was 92.86%, and the specificity was 64.29% in classification, which resulted in the best classification choice (Figure 2B).

### Correlation analysis result

**Figure 3:**
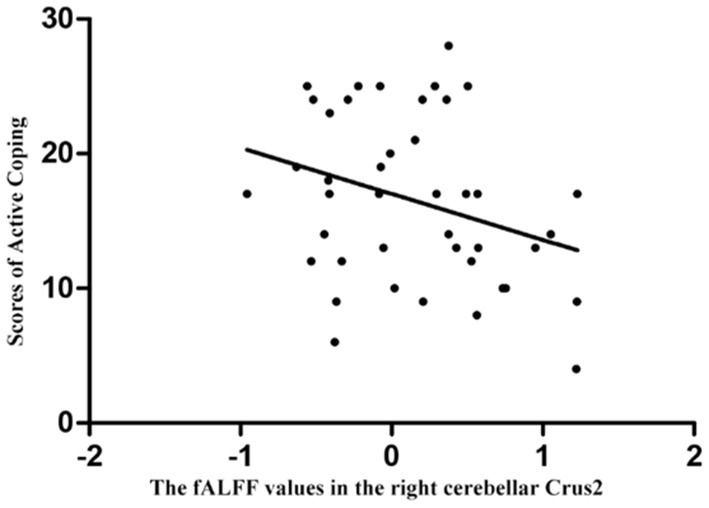
Correlations between abnormal fALFF values and clinical variables. Abnormal fALFF values of the right cerebellar Crus2 were significantly correlated with the active coping scores in the Simplified Coping Style Questionnaire in the patients (*r* = −0.307, *p* = 0.048), and the correlation was no longer significant after the Bonferroni correction.

Supplementary Table S1 showed the details of correlation analysis results. Pearson or Spearman correlation analyses showed that abnormal fALFF values of the right cerebellar Crus2 were significantly correlated with the active coping scores in the SCSQ (*r* = −0.307, *p* = 0.048, df = 41) in the patients (Figure 3), however, the correlation was no longer significant after the Bonferroni correction. There was no correlation between abnormal fALFF and other clinical variables, eye movements, and event-related potential related indicators in the patients. No association between fALFF and clinical variables, eye movements, and event-related potential related indicators was observed in HCs.

## Discussion

The study used the fALFF analysis method to investigate the underlying neuroimaging changes in MDD. The results of the study showed that the fALFF values of the sensorimotor network, including the right middle temporal gyrus, right cerebellar Crus2, left occipital gyrus, and left middle temporal gyrus, were significantly higher compared to HCs, which is consistent with our hypothesis. However, contrary to our hypothesis, the study only found that the abnormal fALFF value of the right cerebellar Crus2 was inversely correlated with the active coping scores in the SCSQ in the patients, and the correlation was no longer significant after the Bonferroni correction. No correlation was observed between abnormal fALFF values and clinical symptoms, neuropsychological tests, eye movements, and event-related potential-related indicators in patients with MDD.

In recent years, the temporal lobe has been a “focal point” of MDD research. The combination of emotional problems and impaired cognitive function is a hallmark of MDD (Fales et al., 2008). At present, a large number of studies have shown that the temporal lobe is closely related to emotional regulation and cognitive functions (selective attention, working memory) (Beauregard et al., 2006; Goulden et al., 2012). A previous study showed that preschoolers with depression had increased FC between the middle temporal gyrus (MTG) and the posterior cingulate cortex (PCC), and this change was associated with a lack of use of active emotion regulation strategies and other behaviors (Gaffrey et al., 2012). Ma et al. (2012) found abnormal FC between MTG and the default-mode Network (DMN) might lead to the negativity of patients' emotional experiences and thinking patterns. In addition to abnormal FC, previous studies have found increased regional homogeneity in the temporal lobe in patients with MDD (Guo et al., 2011; Wu et al., 2011). In addition, Peng et al. (2011) and Kandilarova et al. (2019) found reduced temporal gray matter volume in patients with MDD. Similar to previous studies, our study found that the fALFF value of bilateral MTG was significantly higher than that of HCs. The negative emotion and cognitive impairment in patients with MDD may be partly related to spontaneous neuronal activity in the middle temporal gyrus.

The sensorimotor network is the “sensor” of brain which primarily responsible for sensory input, processing, and forming the sensory experience. The sensorimotor network, including the superior frontal/supplementary motor area, lingual gyrus, and suboccipital/temporal gyrus (Shirer et al., 2012; Chen et al., 2020), is closely connected with other brain networks and participates in important activities together (Stevens et al., 2007; Chang et al., 2013; Doucet et al., 2017; Comstock et al., 2018; Pi et al., 2019). The middle temporal gyrus is a brain region related to the primary sensory pathway and plays an important role in the motor sensory network (Wang et al., 2019), which indicates that the abnormal motor sensory network may also be involved in the negative emotion and cognitive impairment of patients with MDD.

In addition, SVM analysis showed that the fALFF value of the left middle temporal gyrus achieved the highest accuracy of 78.57%, a sensitivity of 92.86%, and a specificity of 64.29% in distinguishing patients from HCs. Considering that in the medical field, both specificity and sensitivity of 70% or more indicate a high degree of confidence (Chen et al., 2021), Therefore, the fALFF value of the left middle temporal gyrus can distinguish patients from HCs, but it is not an ideal indicator because the specificity is slightly low.

The occipital lobe contains most of the anatomical areas of the visual cortex, contributes to visual information processing and communication with the cerebral cortex, and plays a role in the perception and processing of facial emotions (Teng et al., 2018; Li and Wang, 2021). Abnormal neural activity of the occipital gyrus in patients with MDD is involved in abnormal neuropsychological processes such as attentional deficit and bradykinesia (Yu et al., 2017). We found that the fALFF value of the left occipital gyrus was significantly higher in patients with MDD compared with HCs, which is inconsistent with the results of some previous studies. The results of some previous studies mainly found a reduction in the left occipital gyrus activation (Guo et al., 2012; Fan et al., 2013; Zhong et al., 2016; Teng et al., 2018). Liang et al. (2013) found increased ReHo in the left middle occipital gyrus in patients with MDD. A previous study on regional homogeneity in social anxiety disorder showed that increased ReHo in the left middle occipital gyrus may be related to hypervigilance in social anxiety disorder (Qiu et al., 2011), Therefore, Liang et al. speculated that abnormalities in the left occipital gyrus in patients with MDD may be related to memory loss and lack of social skills. Guo et al. (2012) showed decreased occipital lobe activation in patients with MDD, suggesting that depression is related to abnormal interruption of visual information processing. Consistent with our findings, Cheng et al. (2019) found hyperactivity in the left middle occipital gyrus in patients with MDD and hypothesized that this abnormality might be related to compensatory effects related to the state of the left occipital gyrus. Therefore, there may be a compensatory effect on the left occipital gyrus in MDD for the impairment of cognitive processing in patients with MDD.

Many studies have shown that the cerebellum is not only involved in balance and motor control, but also in emotional regulation and cognitive processes such as attention, memory, and suppression of impulsive decision-making (Bugalho et al., 2006; Ravizza et al., 2006; Schmahmann et al., 2007; Lin et al., 2012; Phillips et al., 2015). Cheng et al. (2019) found increased activity in the anterior cerebellar lobe in patients with MDD in remission compared with patients with first-episode, drug-free MDD, and they hypothesized that state-related cerebellar overactivity in the remission group was involved in a compensatory mechanism. Zhang et al. (2022) found that ALFF and fALFF values were significantly increased in the left cerebellum of patients with MDD, which may be related to abnormal emotional processing and disease states. Xiong et al. (2022) found that the spontaneous activity of the cerebellum was enhanced before and after treatment, indicating that such changes may be related to negative thinking and clinical symptoms of MDD. Similarly, Wang et al. (2012) found a significant increase in cerebellar ALFF and fALFF in patients with MDD at rest, and this abnormality may partly explain the emotional abnormalities and cognitive symptoms of depression. Of course, there are some different results. Liu et al. (2014) found that cerebellar ALFF values were significantly reduced in patients compared with HCs, which may be due to disrupted cerebellar to cerebral interactions, resulting in impaired internal environmental adjustment ability, making it difficult for patients to adapt to environmental demands.

In this study, we found that the fALFF value of the right cerebellar Crus2 in patients with MDD was significantly increased, and this increase in fALFF value was negatively correlated with the active coping scores in the SCSQ. We hypothesize that the increased spontaneous neural activity in the cerebellum of patients with MDD may be partly related to the impaired ability of emotional regulation and cognitive processes.

Unfortunately, our study did not find correlations between abnormal fALFF and cognitive function, eye movements, and event-related potential indicators. First, the unmatched age between the two groups might confound the results, although the age served as the covariate. Second, the small sample size and the short disease course of some first-episode patients may account for no correlations. Third, the history of psychiatric medication exposure might alter the spontaneous neural activity, eye movements, and event-related potential in patients with MDD.

There are some shortcomings in this study. First, the sample size of this study is relatively small. Second, we recruited patients who were medication free and those who did not take drugs for at least 2 weeks before enrollment. The influence of psychotropic drugs and the number of episodes on spontaneous brain activity cannot be completely excluded for recurrent patients, which may limit the generalization of the findings. Finally, we scanned the brains of patients with MDD only at baseline, we could not know spontaneous neuronal activity after treatment.

## Conclusion

The study found that compared to HCs, the sensorimotor network, including the right middle temporal gyrus, right cerebellar Crus2, left occipital gyrus, and left middle temporal gyrus, showed enhanced spontaneous neural activity in the patients with MDD. No associations were found between abnormal spontaneous neural activity and clinical variables, eye movements, and event-related potential related indicators in MDD.

## Data availability statement

The raw data supporting the conclusions of this article will be made available by the authors, without undue reservation.

## Ethics statement

The studies involving human participants were reviewed and approved by the Research Ethics Committee of the Third People's Hospital of Foshan. The patients/participants provided their written informed consent to participate in this study.

## Author contributions

QZ, XL, and HY: methodology, data curation, formal analysis, and writing and editing. YW, YO, YY, JL, HL, WW, and XM: conceptualization and data curation. GX and WG: methodology, data curation, writing—review and editing, and funding acquisition. All authors contributed to the article and approved the submitted version.
