# Supplementary material for: Associations between abnormal spontaneous neural activity and clinical variables, eye movements, and event-related potential indicators in major depressive disorder
Source: Front Neurosci. 2023 Jan 11;16:1056868. doi: 10.3389/fnins.2022.1056868 (PMC9875062; doi:10.3389/fnins.2022.1056868)
Supplement: Supplementary file 1 [file Table_1.doc]

Table S1. Correlation analysis results.
	Right Middle Temporal Gyrus	Right Cerebellar Crus2	Left Occipital Gyrus	Left Middle Temporal Gyrus	
R1	r	-.078	-.206	.123	.000	
	P	.632	.203	.448	.999	
	Number of participants	40	40	40	40	
R4	r	-.150	-.129	-.022	.161	
	P	.355	.429	.894	.320	
	Number of participants	40	40	40	40	
R5	r	-.091	-.204	.059	.049	
	P	.579	.206	.716	.762	
	Number of participants	40	40	40	40	
HR¡]times/minute¡^	r	-.024	.091	-.024	-.236	
	P	.883	.576	.883	.143	
	Number of participants	40	40	40	40	
QRS width(ms¡^	r	-.078	.033	-.053	-.274	
	P	.634	.842	.746	.087	
	Number of participants	40	40	40	40	
PR interval¡]ms¡^	r	.194	-.122	-.153	.183	
	P	.229	.455	.347	.260	
	Number of participants	40	40	40	40	
QTc¡]ms¡^	r	-.061	.055	.170	-.113	
	P	.708	.738	.293	.486	
	Number of participants	40	40	40	40	
TG¡]mmol/L¡^	r	.198	-.092	-.096	-.064	
	P	.210	.561	.547	.689	
	Number of participants	42	42	42	42	
CHOL¡]mmol/L¡^	r	.253	-.141	-.021	-.064	
	P	.106	.374	.895	.688	
	Number of participants	42	42	42	42	
HDL¡]mmol/L¡^	r	.118	-.026	.029	.170	
	P	.456	.870	.857	.280	
	Number of participants	42	42	42	42	
LDL¡]mmol/L¡^	r	.226	-.140	-.021	-.162	
	P	.149	.376	.893	.304	
	Number of participants	42	42	42	42	
FBG¡]mmol/L¡^	r	.073	.094	-.140	-.096	
	P	.667	.558	.383	.550	
	Number of participants	41	41	41	41	
TSH3UL¡]mIU/L¡^	r	-.304	-.162	.251	.221	
	P	.050	.305	.108	.098	
	Number of participants	42	42	42	42	
FT3¡]pmol/L¡^	r	.268	-.064	.031	-.017	
	P	.086	.685	.844	.917	
	Number of participants	42	42	42	42	
FT4¡]pmol/L¡^	r	.120	.188	.085	.263	
	P	.449	.233	.593	.093	
	Number of participants	42	42	42	42	
Cortisol¡]nmol/L¡^	r	.062	.089	-.073	.163	
	P	.701	.579	.649	.309	
	Number of participants	41	41	41	41	
Uric acid¡]£gmol/L)	r	-.034	-.137	-.054	-.096	
	P	.831	.392	.737	.550	
	Number of participants	41	41	41	41	
HAMD score	r	-.077	.130	-.102	-.195	
	P	.639	.424	.530	.229	
	Number of participants	40	40	40	40	
HAMA score	r	.083	.099	-.165	-.130	
	P	.610	.543	.308	.424	
	Number of participants	40	40	40	40	
P	r	-.112	-.154	.073	-.037	
	P	.481	.329	.648	.814	
	Number of participants	42	42	42	42	
E	r	.188	-.180	-.060	.042	
	P	.234	.253	.704	.792	
	Number of participants	42	42	42	42	
N	r	-.195	.128	.254	.064	
	P	.217	.418	.105	.686	
	Number of participants	42	42	42	42	
L	r	.137	.212	-.087	.097	
	P	.385	.178	.583	.543	
	Number of participants	42	42	42	42	
EPQ total score 	r	.089	.063	.094	.114	
	P	.575	.691	.554	.471	
	Number of participants	42	42	42	42	
SDSS score	r	.015	.000	.255	.015	
	P	.924	.998	.104	.924	
	Number of participants	42	42	42	42	
SSS_Objective	r	.135	-.188	.050	.126	
	P	.393	.232	.754	.427	
	Number of participants	42	42	42	42	
SSS_Subjective	r	.037	-.137	-.038	.031	
	P	.815	.387	.813	.846	
	Number of participants	42	42	42	42	
SSS_Social	r	.275	-.131	-.096	.002	
	P	.078	.408	.543	.992	
	Number of participants	42	42	42	42	
SSS total score	r	.142	-.188	-.027	.068	
	P	.369	.234	.867	.668	
	Number of participants	42	42	42	42	
Active coping	r	-.081	-.307*	-.173	.023	
	P	.611	.048	.272	.885	
	Number of participants	42	42	42	42	
Negative coping	r	-.110	-.067	-.201	-.217	
	P	.488	.675	.202	.168	
	Number of participants	42	42	42	42	
SCSQ total score	r	-.115	-.259	-.229	-.092	
	P	.469	.097	.145	.562	
	Number of participants	42	42	42	42	
At	r	.143	.111	.114	-.025	
	P	.372	.448	.476	.876	
	Number of participants	41	41	41	41	
Bt	r	.104	.153	.072	.008	
	P	.517	.339	.656	.958	
	Number of participants	41	41	41	41	
Ct	r	.096	.145	.038	.008	
	P	.551	.366	.815	.958	
	Number of participants	41	41	41	41	
(C-B)/A	r	.078	-.040	-.198	-.031	
	P	.628	.803	.216	.850	
	Number of participants	41	41	41	41	
C-2B+100	r	-.077	-.116	-.099	-.005	
	P	.631	.470	.538	.976	
	Number of participants	41	41	41	41	
Ae(missay)	r	.074	-.254	-.119	.122	
	P	.644	.109	.458	.448	
	Number of participants	41	41	41	41	
Ae(correction)	r	.070	-.085	.031	.103	
	P	.663	.597	.845	.523	
	Number of participants	41	41	41	41	
Ae(block)	r	-.097	.222	-.045	-.227	
	P	.546	.163	.779	.153	
	Number of participants	41	41	41	41	
Ae(total)	r	-.003	.001	-.074	-.059	
	P	.983	.993	.643	.712	
	Number of participants	41	41	41	41	
Be(missay)	r	.240	-.021	-.239	.026	
	P	.131	.897	.133	.869	
	Number of participants	41	41	41	41	
Be(correction)	r	.064	-.212	.152	.291	
	P	.692	.184	.344	.065	
	Number of participants	41	41	41	41	
Be(block)	r	-.116	.152	-.060	-.068	
	P	.468	.343	.709	.700	
	Number of participants	41	41	41	41	
Be(total)	r	.059	.001	-.085	-.117	
	P	.712	.997	.596	.468	
	Number of participants	41	41	41	41	
Ce(missay)	r	.153	.127	-.002	.059	
	P	.524	.430	.990	.716	
	Number of participants	41	41	41	41	
Ce(correction)	r	.167	-.156	-.219	.020	
	P	.297	.329	.168	.903	
	Number of participants	41	41	41	41	
Ce(block)	r	-.077	.214	-.288	-.236	
	P	.631	.179	.068	.138	
	Number of participants	41	41	41	41	
Ce(total)	r	.213	.111	-.244	-.085	
	P	.181	.488	.124	.599	
	Number of participants	41	41	41	41	
(C-B)/A	r	.170	.176	-.275	.142	
	P	.289	.271	.082	.377	
	Number of participants	41	41	41	41	
C-2B+100	r	.104	.033	.041	.131	
	P	.520	.839	.797	.416	
	Number of participants	41	41	41	41	
RBANS 1A	r	-.126	-.312	.130	-.073	
	P	.438	.050	.424	.654	
	Number of participants	40	40	40	40	
RBANS 1B	r	-.006	-.040	.091	.086	
	P	.969	.806	.576	.599	
	Number of participants	40	40	40	40	
RBANS 2	r	.017	-.261	.209	.266	
	P	.916	.104	.196	.097	
	Number of participants	40	40	40	40	
RBANS 3	r	.196	.112	-.001	.122	
	P	.225	.490	.997	.454	
	Number of participants	40	40	40	40	
RBANS 4A	r	-.304	-.196	.278	.129	
	P	.056	.226	.082	.426	
	Number of participants	40	40	40	40	
RBANS 4B	r	-.106	-.104	-.080	.154	
	P	.514	.522	.622	.342	
	Number of participants	40	40	40	40	
RBANS 5A	r	-.118	-.240	.071	-.033	
	P	.470	.136	.664	.841	
	Number of participants	40	40	40	40	
RBANS 5B	r	-.216	-.125	.136	.265	
	P	.182	.506	.402	.098	
	Number of participants	40	40	40	40	
RBANS 5C	r	-.022	-.042	.009	.093	
	P	.895	.799	.958	.570	
	Number of participants	40	40	40	40	
RBANS 5D	r	-.056	-.171	.048	-.011	
	P	.729	.292	.767	.947	
	Number of participants	40	40	40	40	
CC	r	.010	.007	.024	.186	
	P	.951	.965	.882	.239	
	Number of participants	42	42	42	42	
RA	r	-.013	.122	.114	-.100	
	P	.936	.442	.470	.530	
	Number of participants	42	42	42	42	
RC	r	.121	.135	-.160	.074	
	P	.444	.392	.311	.644	
	Number of participants	42	42	42	42	
RE	r	-.103	-.062	.175	-.099	
	P	.514	.697	.268	.533	
	Number of participants	42	42	42	42	
RP	r	-.043	-.027	.090	-.109	
	P	.786	.867	.570	.493	
	Number of participants	42	42	42	42	
RPE	r	-.010	.036	.104	-.118	
	P	.952	.822	.510	.458	
	Number of participants	42	42	42	42	
NEF	r	.054	.014	-.225	-.187	
	P	.785	.945	.250	.342	
	Number of participants	28	28	28	28	
RSS1	r	-.173	-.175	.300	.238	
	P	.381	.374	.121	.223	
	Number of participants	28	28	28	28	
RSS2	r	.166	-.054	-.114	.005	
	P	.398	.784	.563	.981	
	Number of participants	28	28	28	28	
RSS(¡×RSS1+RSS2)	r	-.016	-.340	.097	.115	
	P	.934	.076	.624	.561	
	Number of participants	28	28	28	28	
D	r	.130	.119	-.027	-.078	
	P	.509	.548	.892	.691	
	Number of participants	28	28	28	28	
N100	r	-.085	.057	.084	.057	
	P	.618	.739	.623	.735	
	Number of participants	37	37	37	37	
P200	r	-.085	.230	.025	-.049	
	P	.613	.165	.880	.770	
	Number of participants	38	38	38	38	
N200	r	.248	.161	-.078	-.039	
	P	.133	.334	.644	.815	
	Number of participants	38	38	38	38	
P300	r	-.018	.092	.160	.193	
	P	.913	.582	.338	.247	
	Number of participants	38	38	38	38	
